# (*E*)-1-(3,3′-Dimeth­oxy-4′-{[(*E*)-4-nitro­benzyl­idene]amino}-[1,1′-biphen­yl]-4-yl)-*N*-(4-nitro­phen­yl)methanimine

**DOI:** 10.1107/S2414314626002208

**Published:** 2026-03-05

**Authors:** Ouafa Boukhemis, Ahlem Linda Boulkedid, Mehdi Boutebdja, Amel Marir, Mohamed Salah Benlatreche, Erwann Jeanneau

**Affiliations:** a National Biotechnology Research Center (CRBT), Industrial Biotechnology Division, Ali Mendjli New City, UV 03 BP E73, Constantine 25000, Algeria; b Unité de Recherche de Chimie de l'Environnement et Moléculaire Structurale Université de Constantine-1 25000 Constantine Algeria; c Laboratoire de Technologie des Matériaux Avancés École Nationale Polytechnique de Constantine Algeria; d Université Constantine 1, Algeria; e The Environmental Engineering and Technologies Laboratory, Abdelhafid Boussouf Mila University, Algeria; f Centre de Diffractométrie Henri Longchambon Université Claude Bernard Lyon1 5 rue de la Doua 69100 Villeurbanne France; Purdue University, USA

**Keywords:** crystal structure, Schiff base

## Abstract

The title compound, C_28_H_22_N_4_O_6_, crystallizes with one half-mol­ecule in the asymmetric unit centered on a twofold rotation axis located at the midpoint of the central C—C bond. The structure is non-planar overall, with nearly planar anisole-imine and nitro­phenyl-methanimine fragments. In the crystal, a cooperative network of inter­molecular C—H⋯O hydrogen bonds organizes the mol­ecules into layers parallel to the (101) plane, which are further consolidated by offset π–π stacking between centrosymmetrically related phenyl rings and C—H⋯π inter­actions. Collectively, these non-covalent inter­actions contribute to the cohesion of the three-dimensional supra­molecular framework.

## Abstract 2

The title compound crystallizes in the monoclinic space group *I*2/*a* with one half-mol­ecule in the asymmetric unit. The structure exhibits non-planar Schiff base moieties, extended π-conjugation, and a supra­molecular framework consolidated by C—H⋯O, π–π stacking, and C—H⋯π inter­actions forming layers parallel to the (101) plane.

## Graphical abstract

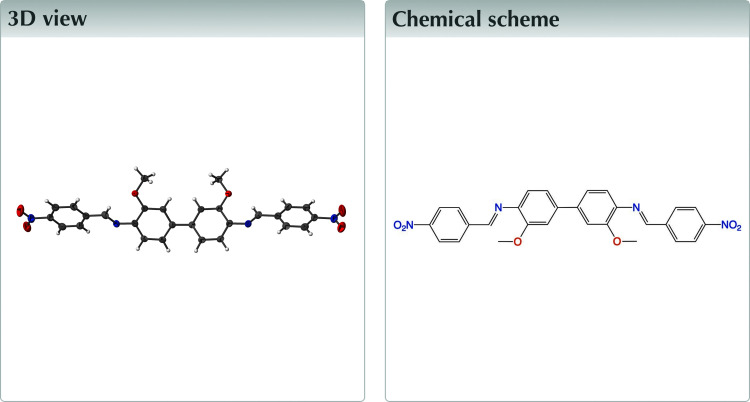

## Structure description

**Figure 1:**
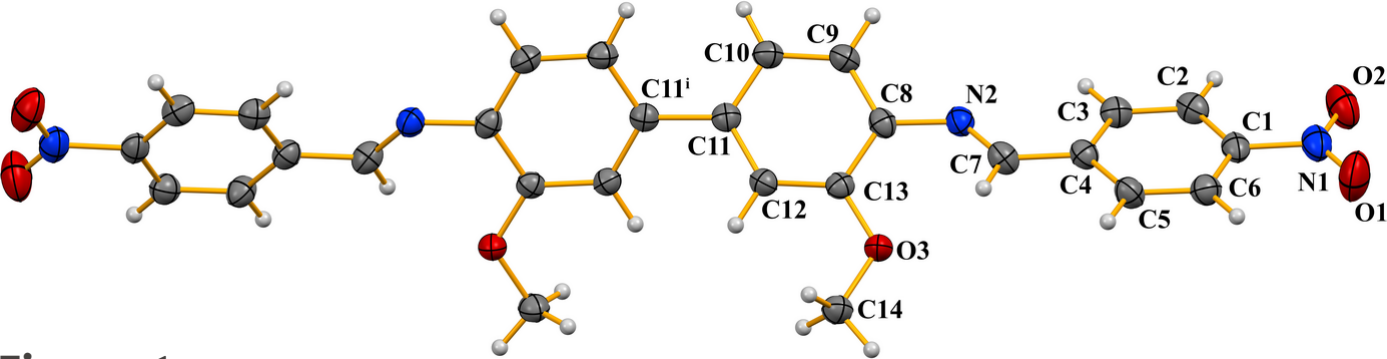
View of the title compound with the atom-numbering scheme. Displacement ellipsoids for non-H atoms are drawn at the 50% probability level. Symmetry code (i): 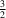 − *x*, 1 − *y*, 1 − *z*.

**Figure 2:**
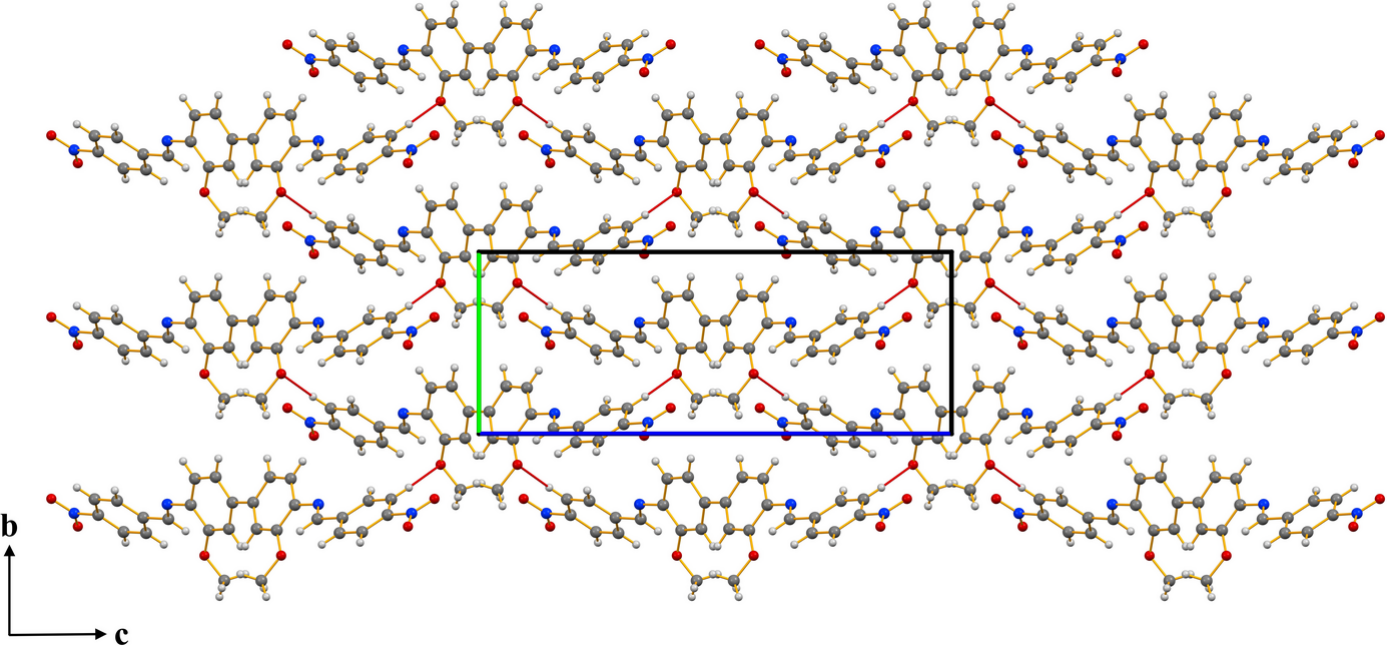
Extended packing arrangement viewed in the [010] direction of the unit cell showing C—H⋯O inter­molecular inter­actions (red line).

**Figure 3:**
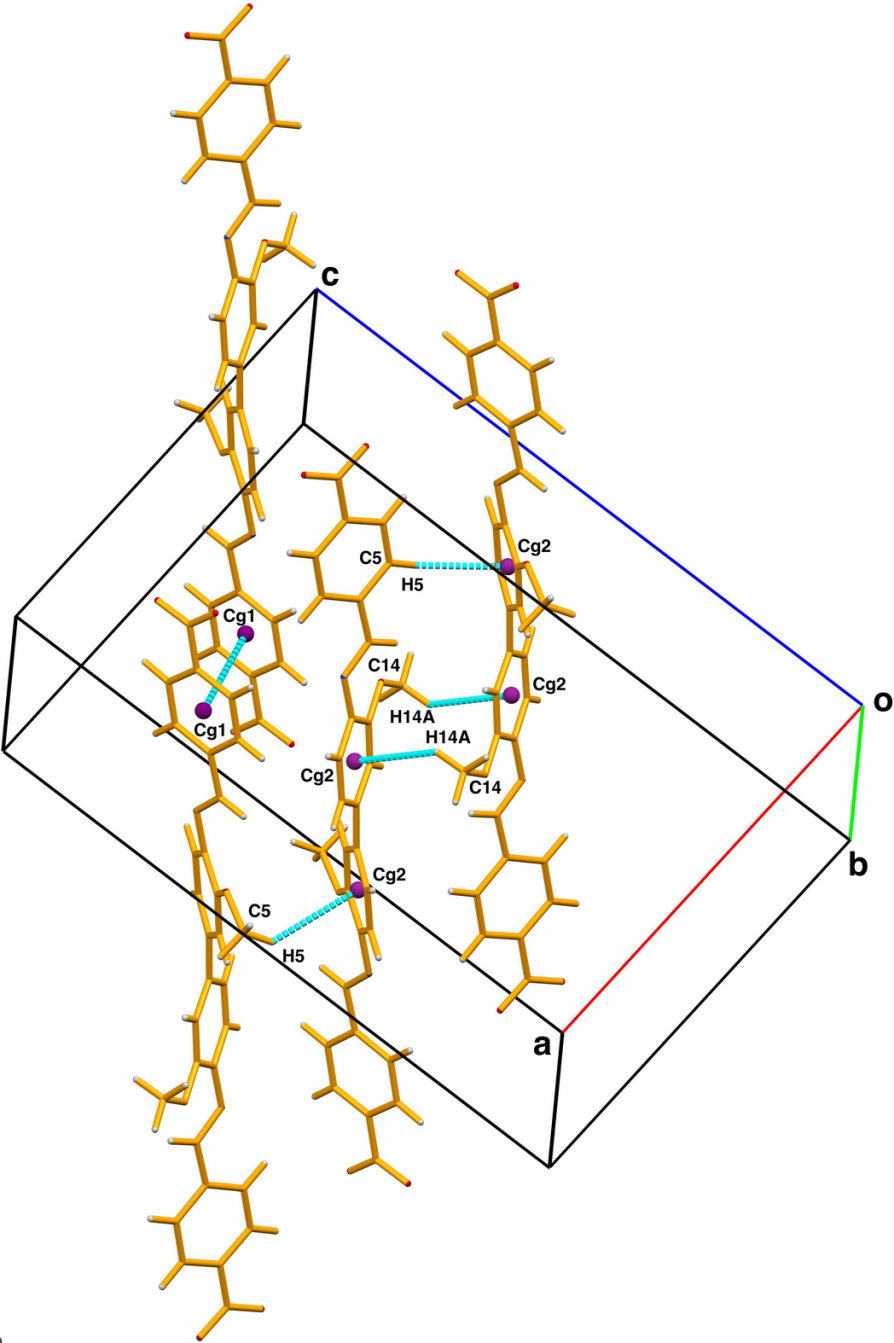
Graphical view of C—H⋯π and π-π stacking inter­actions.

The title Schiff base was synthesized as part of a broader search for multifunctional imine-based mol­ecules, whose tunable π-conjugation underpins applications in catalysis, sensing, materials science and medicinal chemistry. The title compound crystallizes in the monoclinic space group *I*2/*a*, with one half-mol­ecule in the asymmetric unit. The mol­ecule is centered on a crystallographic twofold rotation axis located at the midpoint of the C11—C11^i^ bond of the amine mol­ecule [symmetry code: (i) 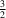 − *x*, 1 − *y*, 1 − *z*] (Fig. 1 ▸). The mol­ecule is not planar. Within the asymmetric unit, the two Schiff base moieties are also non-planar, whereas the anisole-imine and nitro­phenyl-methanimine are each individually nearly planar (C1—C6 and N, O1, O2, C7 are planar with a maximum deviation of 0.045 (2) Å for the azomethine carbon atom C7; and C8–C13, N2, O3 and C14 are planar with a maximum deviation of 0.148 (1) Å for the azomethine nitro­gen atom N2). In the two six-membered rings, the largest distance between atom and the mean plane is 0.005 (2) Å, and 0.018 (2) Å in the nearly planar parts, respectively. Bond length analysis indicates an extended network of π bonds across the mol­ecule, consistent with pronounced electronic delocalization. The N2—C7 bond length of 1.266 (2) Å is characteristic of a C=N double bond, confirming significant π character within the imine fragment. The C7—N2—C8 bond angle of 122.82 (14)° shows that atom N2 adopts an essentially trigonal–planar geometry, in agreement with *sp*^2^ hybridization and conjugation with the adjacent aromatic ring system. The imine unit adopts an almost perfectly *trans* arrangement, with the atoms around the C=N bond being essentially coplanar as indicated by the C8—N2—C7—C4 torsion angle of −179.82(14°). The bond distances and angles are normal and are in good agreement with those in analogous structures (Hernández Téllez *et al.*, 2025 ▸; Adam *et al.*, 2015 ▸; Madhuprasad *et al.*, 2014 ▸). The dihedral angle between the aromatic rings in the asymmetric unit is 35.68 (7)° and is largely controlled by the cooperative effect of the intra­molecular C7—H7⋯O3 and the inter­molecular C2—H2⋯O3 hydrogen bonds (Table 1 ▸). The latter inter­action links neighboring mol­ecules, forming layers parallel to the (101) plane (Fig. 2 ▸). In the crystal, slightly offset π–π stacking inter­actions are present between centrosymmetrically related phenyl rings with a *Cg*1⋯*Cg*1(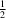 − *x*, 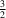 − *y*, 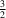 − *z*) separation of 3.5385 (10) Å, slippage = 1.235 Å, where *Cg*1 is the centroid of the C1–C6 ring. The overall crystal cohesion is reinforced by C—H⋯π inter­actions involving C5—H5⋯*Cg*2(−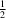 + *x*, 1 − *y*, *z*) and C14—H14*A*⋯*Cg*2(1 − *x*, 1 − *y*, 1 − *z*) inter­actions, with H⋯*Cg* separations of 3.4898 (18) and 3.6757 (17) Å, respectively, and C—H⋯*Cg* angles of 140 and 129°, respectively, where *Cg*2 is the centroid of the C8–C13 ring (Fig. 3 ▸). Collectively, these non-covalent inter­actions contribute to the cohesion of the three-dimensional supra­molecular framework.

## Synthesis and crystallization

An ethano­lic solution of 4-nitro­salicyl­aldehyde (0.02 mmol, 3.34 mg) was added dropwise to a methano­lic solution of *O*-dianisidine (0.01 mmol, 2.44 mg) under constant magnetic stirring at room temperature. The reaction mixture was stirred for 1 h, during which the solution gradually turned orange. The product was obtained as brown crystals suitable for X-ray diffraction analysis after slow evaporation of the solvent, washed with cold ethanol, and dried in a desiccator. Yield: 76.24%. m.p 529.15 K.

FTIR (ATR, cm^−1^): 3100–3000 (*w*) (aromatic/*sp*^2^ C—H stretching), 2924 (*w*) (asymmetric aliphatic C—H stretching), 2857 (*w*) (symmetric aliphatic C—H stretching), 1687 (*w*)–1630 (*vs*) (azomethine stretching vibration C=N), 1599 (*s*) (N—O asymmetric stretching), 1371 (*w*) (N—O symmetric stretching).

## Refinement

Crystal data, data collection and structure refinement details are summarized in Table 2 ▸.

## Supplementary Material

Crystal structure: contains datablock(s) global, I. DOI: 10.1107/S2414314626002208/zl4090sup1.cif

Structure factors: contains datablock(s) I. DOI: 10.1107/S2414314626002208/zl4090Isup3.hkl

The ATRFTIR spectrum. DOI: 10.1107/S2414314626002208/zl4090sup4.docx

Supporting information file. DOI: 10.1107/S2414314626002208/zl4090Isup4.cml

CCDC reference: 2487703

Additional supporting information:  crystallographic information; 3D view; checkCIF report

## Figures and Tables

**Table 1:** Hydrogen-bond geometry (Å, °)

*D*—H⋯*A*	*D*—H	H⋯*A*	*D*⋯*A*	*D*—H⋯*A*
C2—H2⋯O3^i^	0.95	2.59	3.3833 (19)	141
C7—H7⋯O3	0.95	2.25	2.7783 (19)	114

Symmetry code: (i) 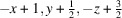.

**Table 2:** Experimental details

Crystal data	
Chemical formula	C_28_H_22_N_4_O_6_
*M*_r_	510.49
Crystal system, space group	Monoclinic, *I*2/*a*
Temperature (K)	150
*a*, *b*, *c* (Å)	14.2099 (14), 8.0341 (8), 20.899 (2)
β (°)	92.171 (9)
*V* (Å^3^)	2384.2 (4)
*Z*	4
Radiation type	Cu *K*α
μ (mm^−1^)	0.85
Crystal size (mm)	0.28 × 0.15 × 0.07

Data collection	
Diffractometer	Xcalibur, Atlas, Gemini ultra
Absorption correction	Multi-scan (*CrysAlis PRO*; Rigaku OD, 2022 ▸)
*T*_min_, *T*_max_	0.729, 1.000
No. of measured, independent and observed [*I* > 2σ(*I*)] reflections	9789, 2120, 1821
*R*_int_	0.038
(sin θ/λ)_max_ (Å^−1^)	0.597

Refinement	
*R*[*F*^2^ > 2σ(*F*^2^)], *wR*(*F*^2^), *S*	0.042, 0.121, 1.06
No. of reflections	2120
No. of parameters	173
H-atom treatment	H-atom parameters constrained
Δρ_max_, Δρ_min_ (e Å^−3^)	0.19, −0.23

Computer programs: *CrysAlis PRO* (Rigaku OD, 2022 ▸), *OLEX2.solve* (Dolomanov *et al.*, 2009 ▸), *SHELXL2019/3* (Sheldrick, 2015 ▸) and *OLEX2* (Dolomanov *et al.*, 2009 ▸).

## full crystallographic data

### (E)-1-(3,3'-Dimethoxy-4'-{[(E)-4-nitrobenzylidene]amino}-[1,1'-biphenyl]-4-yl)-N-(4-nitrophenyl)methanimine Crystal data

**Table:**

C_28_H_22_N_4_O_6_	*F*(000) = 1064
*M**_r_* = 510.49	*D*_x_ = 1.422 Mg m^−^^3^
Monoclinic, *I*2/*a*	Cu *K*α radiation, λ = 1.54184 Å
Hall symbol: -I 2ya	Cell parameters from 5072 reflections
*a* = 14.2099 (14) Å	θ = 4.1–66.9°
*b* = 8.0341 (8) Å	µ = 0.85 mm^−^^1^
*c* = 20.899 (2) Å	*T* = 150 K
β = 92.171 (9)°	Plate, brown
*V* = 2384.2 (4) Å^3^	0.28 × 0.15 × 0.07 mm
*Z* = 4	

### (E)-1-(3,3'-Dimethoxy-4'-{[(E)-4-nitrobenzylidene]amino}-[1,1'-biphenyl]-4-yl)-N-(4-nitrophenyl)methanimine Data collection

**Table:**

Xcalibur, Atlas, Gemini ultra diffractometer	2120 independent reflections
Mirror monochromator	1821 reflections with *I* > 2σ(*I*)
Detector resolution: 10.4685 pixels mm^-1^	*R*_int_ = 0.038
ω scans	θ_max_ = 67.0°, θ_min_ = 4.2°
Absorption correction: multi-scan (CrysAlisPro; Rigaku OD, 2022)	*h* = −16→16
*T*_min_ = 0.729, *T*_max_ = 1.000	*k* = −9→9
9789 measured reflections	*l* = −24→20

### (E)-1-(3,3'-Dimethoxy-4'-{[(E)-4-nitrobenzylidene]amino}-[1,1'-biphenyl]-4-yl)-N-(4-nitrophenyl)methanimine Refinement

**Table:**

Refinement on *F*^2^	Primary atom site location: dual
Least-squares matrix: full	Hydrogen site location: inferred from neighbouring sites
*R*[*F*^2^ > 2σ(*F*^2^)] = 0.042	H-atom parameters constrained
*wR*(*F*^2^) = 0.121	W = 1/[Σ^2^(*F*O^2^) + (0.068*P*)^2^ + 1.2673*P*] WHERE *P* = (*F*O^2^ + 2*F*C^2^)/3
*S* = 1.06	(Δ/σ)_max_ = 0.001
2120 reflections	Δρ_max_ = 0.19 e Å^−^^3^
173 parameters	Δρ_min_ = −0.23 e Å^−^^3^
0 restraints	Absolute structure: All H atoms were placed in geometrically idealized positions and constrained to ride on their parent atoms, with C—H distances of 0.95–0.98 Å with *U*_iso_(H) values of 1.2 or 1.5 *U*_eq_ of the parent atoms.

### (E)-1-(3,3'-Dimethoxy-4'-{[(E)-4-nitrobenzylidene]amino}-[1,1'-biphenyl]-4-yl)-N-(4-nitrophenyl)methanimine Special details

**Table:**

Geometry. Bond distances, angles etc. have been calculated using the rounded fractional coordinates. All su's are estimated from the variances of the (full) variance-covariance matrix. The cell esds are taken into account in the estimation of distances, angles and torsion angles

### (E)-1-(3,3'-Dimethoxy-4'-{[(E)-4-nitrobenzylidene]amino}-[1,1'-biphenyl]-4-yl)-N-(4-nitrophenyl)methanimine Fractional atomic coordinates and isotropic or equivalent isotropic displacement parameters (Å^2^)

**Table:**

	*x*	*y*	*z*	*U*_iso_*/*U*_eq_	
O1	0.08857 (11)	0.4892 (2)	0.84954 (7)	0.0664 (6)	
O2	0.18453 (10)	0.64173 (19)	0.90565 (6)	0.0538 (5)	
O3	0.53245 (7)	0.32934 (13)	0.58125 (5)	0.0311 (3)	
N1	0.16324 (11)	0.56187 (19)	0.85733 (7)	0.0409 (5)	
N2	0.50068 (9)	0.61140 (16)	0.66003 (6)	0.0305 (4)	
C1	0.23150 (11)	0.55294 (19)	0.80633 (7)	0.0313 (5)	
C2	0.31525 (12)	0.6393 (2)	0.81402 (7)	0.0330 (5)	
C3	0.37976 (11)	0.6307 (2)	0.76630 (7)	0.0318 (5)	
C4	0.35916 (11)	0.53793 (19)	0.71097 (7)	0.0297 (5)	
C5	0.27422 (11)	0.4534 (2)	0.70499 (8)	0.0339 (5)	
C6	0.20921 (11)	0.4594 (2)	0.75260 (8)	0.0345 (5)	
C7	0.42422 (11)	0.5304 (2)	0.65778 (8)	0.0327 (5)	
C8	0.56487 (10)	0.60829 (19)	0.61024 (7)	0.0280 (4)	
C9	0.61697 (11)	0.75257 (19)	0.60148 (7)	0.0298 (5)	
C10	0.68751 (11)	0.76153 (19)	0.55758 (7)	0.0298 (5)	
C11	0.71028 (10)	0.62133 (19)	0.52197 (7)	0.0271 (4)	
C12	0.65722 (11)	0.47610 (18)	0.52939 (7)	0.0272 (4)	
C13	0.58502 (10)	0.46875 (18)	0.57190 (7)	0.0261 (4)	
C14	0.54558 (11)	0.19137 (19)	0.53924 (7)	0.0331 (5)	
H2	0.32825	0.70356	0.85148	0.0400*	
H3	0.43814	0.68782	0.77112	0.0380*	
H5	0.26035	0.39003	0.66742	0.0410*	
H6	0.15118	0.40094	0.74839	0.0410*	
H7	0.40857	0.46390	0.62132	0.0390*	
H9	0.60377	0.84807	0.62640	0.0360*	
H10	0.72033	0.86310	0.55175	0.0360*	
H12	0.67113	0.38055	0.50469	0.0330*	
H14A	0.53360	0.22693	0.49482	0.0500*	
H14B	0.50174	0.10202	0.54958	0.0500*	
H14C	0.61042	0.15064	0.54454	0.0500*	

### (E)-1-(3,3'-Dimethoxy-4'-{[(E)-4-nitrobenzylidene]amino}-[1,1'-biphenyl]-4-yl)-N-(4-nitrophenyl)methanimine Atomic displacement parameters (Å^2^)

**Table:**

	*U*^11^	*U*^22^	*U*^33^	*U*^12^	*U*^13^	*U*^23^
O1	0.0501 (9)	0.0891 (12)	0.0617 (9)	−0.0142 (8)	0.0233 (7)	−0.0021 (8)
O2	0.0611 (9)	0.0675 (9)	0.0334 (7)	0.0152 (7)	0.0106 (6)	−0.0032 (6)
O3	0.0330 (6)	0.0246 (5)	0.0364 (6)	−0.0020 (4)	0.0089 (4)	−0.0027 (4)
N1	0.0429 (9)	0.0456 (8)	0.0348 (8)	0.0107 (7)	0.0093 (6)	0.0070 (7)
N2	0.0283 (7)	0.0316 (7)	0.0317 (7)	0.0034 (5)	0.0039 (5)	0.0007 (5)
C1	0.0335 (8)	0.0320 (8)	0.0287 (8)	0.0090 (6)	0.0054 (6)	0.0059 (6)
C2	0.0383 (9)	0.0329 (8)	0.0274 (7)	0.0048 (6)	−0.0034 (6)	−0.0012 (6)
C3	0.0297 (8)	0.0330 (8)	0.0323 (8)	0.0014 (6)	−0.0024 (6)	0.0011 (6)
C4	0.0286 (8)	0.0294 (8)	0.0312 (8)	0.0041 (6)	0.0012 (6)	−0.0006 (6)
C5	0.0317 (8)	0.0341 (9)	0.0360 (8)	0.0011 (6)	0.0022 (6)	−0.0083 (7)
C6	0.0301 (8)	0.0320 (8)	0.0414 (9)	0.0006 (6)	0.0020 (7)	0.0003 (7)
C7	0.0305 (8)	0.0359 (8)	0.0315 (8)	0.0043 (7)	−0.0004 (6)	−0.0052 (7)
C8	0.0261 (7)	0.0313 (8)	0.0265 (7)	0.0047 (6)	0.0001 (6)	−0.0004 (6)
C9	0.0311 (8)	0.0263 (8)	0.0319 (8)	0.0025 (6)	0.0004 (6)	−0.0039 (6)
C10	0.0300 (8)	0.0252 (8)	0.0342 (8)	−0.0006 (6)	−0.0005 (6)	−0.0012 (6)
C11	0.0267 (7)	0.0284 (8)	0.0261 (7)	0.0011 (6)	−0.0012 (6)	0.0012 (6)
C12	0.0292 (7)	0.0254 (7)	0.0271 (7)	0.0022 (6)	0.0008 (6)	−0.0010 (6)
C13	0.0248 (7)	0.0254 (7)	0.0278 (7)	0.0011 (6)	−0.0009 (6)	0.0021 (6)
C14	0.0383 (9)	0.0282 (8)	0.0330 (8)	−0.0032 (6)	0.0037 (6)	−0.0025 (6)

### (E)-1-(3,3'-Dimethoxy-4'-{[(E)-4-nitrobenzylidene]amino}-[1,1'-biphenyl]-4-yl)-N-(4-nitrophenyl)methanimine Geometric parameters (Å, º)

**Table:**

O1—N1	1.217 (2)	C9—C10	1.386 (2)
O2—N1	1.225 (2)	C10—C11	1.395 (2)
O3—C13	1.3645 (18)	C11—C12	1.401 (2)
O3—C14	1.4306 (18)	C11—C11^i^	1.482 (2)
N1—C1	1.470 (2)	C12—C13	1.384 (2)
N2—C7	1.266 (2)	C2—H2	0.9500
N2—C8	1.4097 (19)	C3—H3	0.9500
C1—C2	1.382 (2)	C5—H5	0.9500
C1—C6	1.378 (2)	C6—H6	0.9500
C2—C3	1.382 (2)	C7—H7	0.9500
C3—C4	1.397 (2)	C9—H9	0.9500
C4—C5	1.386 (2)	C10—H10	0.9500
C4—C7	1.474 (2)	C12—H12	0.9500
C5—C6	1.384 (2)	C14—H14A	0.9800
C8—C9	1.391 (2)	C14—H14B	0.9800
C8—C13	1.414 (2)	C14—H14C	0.9800
			
C13—O3—C14	117.60 (11)	O3—C13—C8	116.49 (13)
O1—N1—O2	123.28 (16)	O3—C13—C12	123.39 (13)
O1—N1—C1	118.41 (14)	C8—C13—C12	120.08 (13)
O2—N1—C1	118.32 (15)	C1—C2—H2	121.00
C7—N2—C8	122.82 (14)	C3—C2—H2	121.00
N1—C1—C2	118.71 (13)	C2—C3—H3	120.00
N1—C1—C6	118.68 (14)	C4—C3—H3	120.00
C2—C1—C6	122.62 (14)	C4—C5—H5	119.00
C1—C2—C3	118.88 (14)	C6—C5—H5	119.00
C2—C3—C4	120.01 (15)	C1—C6—H6	121.00
C3—C4—C5	119.36 (14)	C5—C6—H6	121.00
C3—C4—C7	121.89 (14)	N2—C7—H7	120.00
C5—C4—C7	118.73 (14)	C4—C7—H7	120.00
C4—C5—C6	121.38 (15)	C8—C9—H9	119.00
C1—C6—C5	117.76 (15)	C10—C9—H9	119.00
N2—C7—C4	120.86 (15)	C9—C10—H10	120.00
N2—C8—C9	116.41 (13)	C11—C10—H10	120.00
N2—C8—C13	125.67 (13)	C11—C12—H12	119.00
C9—C8—C13	117.77 (13)	C13—C12—H12	119.00
C8—C9—C10	122.15 (14)	O3—C14—H14A	109.00
C9—C10—C11	119.96 (14)	O3—C14—H14B	109.00
C10—C11—C12	118.48 (13)	O3—C14—H14C	109.00
C10—C11—C11^i^	121.88 (14)	H14A—C14—H14B	109.00
C11^i^—C11—C12	119.63 (13)	H14A—C14—H14C	109.00
C11—C12—C13	121.46 (13)	H14B—C14—H14C	109.00
			
C14—O3—C13—C8	174.58 (12)	C5—C4—C7—N2	176.96 (15)
C14—O3—C13—C12	−7.9 (2)	C4—C5—C6—C1	0.2 (2)
O1—N1—C1—C2	178.07 (16)	N2—C8—C9—C10	174.91 (14)
O1—N1—C1—C6	−1.6 (2)	C13—C8—C9—C10	−0.9 (2)
O2—N1—C1—C2	−2.2 (2)	N2—C8—C13—O3	5.0 (2)
O2—N1—C1—C6	178.16 (15)	N2—C8—C13—C12	−172.64 (14)
C8—N2—C7—C4	−179.82 (14)	C9—C8—C13—O3	−179.61 (13)
C7—N2—C8—C9	148.63 (15)	C9—C8—C13—C12	2.7 (2)
C7—N2—C8—C13	−36.0 (2)	C8—C9—C10—C11	−2.1 (2)
N1—C1—C2—C3	179.75 (14)	C9—C10—C11—C12	3.2 (2)
C6—C1—C2—C3	−0.6 (2)	C9—C10—C11—C11^i^	−175.41 (14)
N1—C1—C6—C5	179.64 (14)	C10—C11—C12—C13	−1.4 (2)
C2—C1—C6—C5	0.0 (2)	C11^i^—C11—C12—C13	177.27 (14)
C1—C2—C3—C4	1.0 (2)	C10—C11—C11^i^—C10^i^	−36.0 (2)
C2—C3—C4—C5	−0.8 (2)	C10—C11—C11^i^—C12^i^	145.35 (15)
C2—C3—C4—C7	177.62 (15)	C12—C11—C11^i^—C10^i^	145.35 (15)
C3—C4—C5—C6	0.2 (2)	C12—C11—C11^i^—C12^i^	−33.3 (2)
C7—C4—C5—C6	−178.29 (15)	C11—C12—C13—O3	−179.10 (13)
C3—C4—C7—N2	−1.5 (2)	C11—C12—C13—C8	−1.6 (2)

Symmetry code: (i) −*x*+3/2, *y*, −*z*+1.

### (E)-1-(3,3'-Dimethoxy-4'-{[(E)-4-nitrobenzylidene]amino}-[1,1'-biphenyl]-4-yl)-N-(4-nitrophenyl)methanimine Hydrogen-bond geometry (Å, º)

**Table:**

*D*—H···*A*	*D*—H	H···*A*	*D*···*A*	*D*—H···*A*
C2—H2···O3^ii^	0.95	2.59	3.3833 (19)	141
C7—H7···O3	0.95	2.25	2.7783 (19)	114

Symmetry code: (ii) −*x*+1, *y*+1/2, −*z*+3/2.
