# Supplementary material for: (E)-1-(3,3′-Dimeth­oxy-4′-{[(E)-4-nitro­benzyl­idene]amino}-[1,1′-biphen­yl]-4-yl)-N-(4-nitro­phen­yl)methanimine
Source: IUCrdata. 2026 Mar 5;11(Pt 3):x260220. doi: 10.1107/S2414314626002208 (PMC13055940; doi:10.1107/S2414314626002208)
Supplement: Supplementary file 3 [file x-11-x260220-sup4.docx]

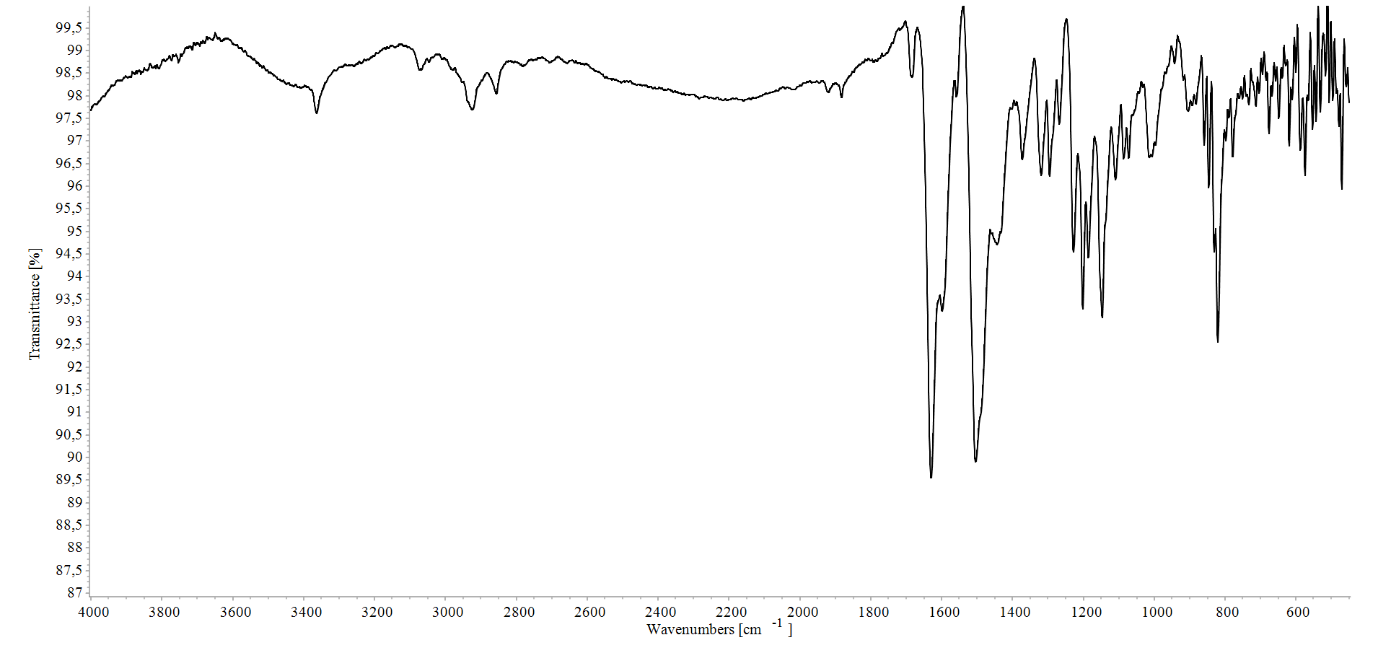


**Figure1.** ATR–FTIR spectrum of the synthesized azomethine (Schiff base) bearing nitro substituents.
